# Supplementary material for: Nutritional Intervention for the Elderly during Chemotherapy: A Systematic Review
Source: Cancers (Basel). 2024 Aug 9;16(16):2809. doi: 10.3390/cancers16162809 (PMC11352472; doi:10.3390/cancers16162809)
Supplement: Supplementary file 1 [file cancers-16-02809-s001.zip › cancers-3136603-supplementary.pdf]

Nutritional intervention for the elderly during chemotherapy: a systematic review

Author	Trial registration number	Sponsorship or funding	Blinding
**Studies evaluating the effect of multimodal intervention**			
Bourdel-Marchasson et al [25]	NCT00459589 (ClinicalTrials.gov)	The study was supported by the National Hospital Program of Clinical Research (Programme Hospitalier de Recherche Clinique 2006) (46%), La Ligue contre le cancer (52%) and AMGEN (2%) and sponsored by the university hospital of Bordeaux (CHU Bordeaux).	Open label trial, participants and investigators were aware of assignment
**Studies evaluating the effect of whey protein supplementation**			
Cereda et al [26]	NCT02065726 (ClinicalTrials.gov)	The study was supported by the Fondazione IRCCS Policlinico San Matteo, partially by the Italian Ministry of Health (project code RF-2011-02351315) and Ricerca Corrente grant no. 08067617, and by Difass International srl. (provision of WPI).	Open label trial, both participants and outcomes assessors were aware of the treatment assignment. The study statistician was blinded to treatment assignment.
**Studies evaluating the effect of amino acids supplementation**			
Katada et al [17]	UMIN000007609 (UMIN Clinical Trials Registry)	None	Blinding of endoscopic-outcomes assessors
D.P. Soares et al [18]	RBR-7ycfcg (The Brazilian registry of Clinical Trials (ReBEC))	None	Blinding of participants
Toyomasu et al [19]	Not registered	None	Open label trial, participants and investigators were aware of assignment
Tanaka et al, 2016 [20]	UMIN000008338 (UMIN Clinical Trials Registry)	Not Available	Open label trial, participants and investigators were aware of assignment
Khemissa et al [22]	NCT00455247 (ClinicalTrials.gov)	The study was funded by Nestle Health Science.	Blinding of participants, support staff and outcomes assessors
Okada et al [21]	UMIN000004898 (UMIN Clinical Trials Registry)	None	Open label trial, participants and investigators were aware of assignment
Tanaka et al, 2021 [24]	UMIN000025412 (UMIN Clinical Trials Registry)	The study was supported by EA Pharma Co., Ltd.	Neither the patients nor investigators were blinded to treatment; outcomes assessors (two specialists from the central review system who assessed the incidence of grade> 2 oral mucositis) were blinded to the treatment assignment
**Studies evaluating the effect of fish oil omega 3 enriched oral supplements**			
Shirai et al [23]	Not registered	Not Available	Open label trial, participants and investigators were aware of assignment

Supplementary table S1: quality of included studies.

Author	Sample size					
	Total	Control group	Intervention group	Withdrawal in control group	Withdrawal in intervention group	Total withdrawal
**Studies evaluating the effect of multimodal intervention**						
Bourdel-Marchasson et al[25]	336	167	169	5	0	5
**Studies evaluating the effect of whey protein supplementation**						
Cereda et al [26]	166	84	82	15	16	31
**Studies evaluating the effect of amino acids supplementation**						
Katada et al [17]	71	36	35	5	4	9
D.P. Soares et al [18]	36	18	18	8	12	20
Toyomasu et al [19]	22	11	11	0	0	0
Tanaka et al, 2016 [20]	30	10	10	0	0	0
Khemissa et al [22]	201	99	102	NA	NA	9
Okada et al [21]	20	10	10	1	1	2
Tanaka et al, 2021[24]	113	58	55	1	3	4
**Studies evaluating the effect of fish oil omega 3 enriched oral supplements**						
Shirai et al [23]	128	91	37	0	51	51

Supplementary table S2: sample size of included studies. NA: not available
